# Shedding Light on SARS-CoV-2, COVID-19, COVID-19 Vaccination, and Auditory Symptoms: Causality or Spurious Conjunction?

**DOI:** 10.3389/fpubh.2022.837513

**Published:** 2022-02-22

**Authors:** Gabrielle H. Saunders, Eldre Beukes, Kai Uus, Christopher J. Armitage, Jack Kelly, Kevin J. Munro

**Affiliations:** ^1^Manchester Centre for Audiology and Deafness, University of Manchester, Manchester, United Kingdom; ^2^Vision and Hearing Sciences Research Centre, Anglia Ruskin University, Cambridge, United Kingdom; ^3^Manchester Centre for Health Psychology, University of Manchester, Manchester, United Kingdom; ^4^National Institute for Health Research (NIHR) Greater Manchester Patient Safety Translational Research Centre, Manchester, United Kingdom; ^5^Manchester University National Health Service (NHS) Foundation Trust, Manchester Academic Health Science Centre, Manchester, United Kingdom; ^6^Centre for Biostatistics, University of Manchester, Manchester, United Kingdom

**Keywords:** SARS-CoV-2, COVID-19, hearing, tinnitus, nocebo effect, self-report, recall bias, COVID-19 vaccine

## Abstract

There are reports of associations between SARS-CoV2, COVID-19, COVID-19 vaccines, and auditory symptoms (hearing difficulty, tinnitus). However, most studies have relied on self-report and lack baseline and/or non-COVID control groups. This makes it problematic to differentiate if symptoms are associated with SARS-CoV2, COVID-19, the vaccine, psychosocial factors or recall bias. In this study, we differentiate these by comparing hearing and tinnitus survey data collected pre- and during the pandemic. The survey conducted during the pandemic asked about the onset and change in three types of symptom. Type One—known association (loss of smell, memory/concentration issues, persistent fatigue), Type Two—indeterminate association (auditory symptoms), and Type Three—no established association with COVID-19 (toothache). We hypothesized that if auditory symptoms are directly associated with COVID-19, their onset and change would be similar to Type One symptoms, but if indirectly associated (reflecting psychosocial factors and/or recall bias) would be more similar to Type Three symptoms. Of the 6,881 individuals who responded, 6% reported confirmed COVID-19 (positive test), 11% probably had COVID-19, and 83% reported no COVID-19. Those with confirmed or probable COVID-19 more commonly reported new and/or worsened auditory symptoms than those not reporting COVID-19. However, this does not imply causality because: (1) new auditory symptoms coincided with COVID-19 illness among just 1/3 of those with confirmed or probable COVID-19, and another 1/3 said their symptoms started before the pandemic—despite reporting no symptoms in the pre-pandemic survey. (2) >60% of individuals who had COVID-19 said it had affected their Type 3 symptoms, despite a lack of evidence linking the two. (3) Those with confirmed COVID-19 reported more Type 1 symptoms, but reporting of Type 2 and Type 3 symptoms did not differ between those with confirmed COVID-19 and those without COVID-19, while those who probably had COVID-19 most commonly reported these symptom types. Despite more reports of auditory symptoms in confirmed or probable COVID-19, there is inconsistent reporting, recall bias, and possible nocebo effects. Studies that include appropriate control groups and use audiometric measures in addition to self-report to investigate change in auditory symptoms relative to pre-COVID-19 are urgently needed.

## Introduction

The World Health Organization declared COVID-19, the disease caused by SARS-CoV-2, a pandemic on 11 March 2020 (1). As of 30 November 2021, the number of reported cases exceeds 268 million worldwide, with more than 5.25 million deaths (https://www.worldmeters.info/coronavirus/accessed 9th December 2021). The symptoms and severity of COVID-19 vary from asymptomatic to severe or fatal (2).

It is well-established that some viral infections cause hearing loss (3), and it has been found that SARS-CoV-2 can infect the inner ear (4). This awareness is reflected in the COVID-19 guidelines from the UK National Institute for Health and Care Excellence (5), which reports that tinnitus, an audiovestibular symptom, can be a common ongoing complaint after COVID-19.

Evidence for the association of audiovestibular symptoms relating to confirmed cases of COVID-19 has been evaluated in several systematic reviews and meta-analyses (6–10). These meta-analyses report pooled prevalence estimates of between 3.1% [95% confidence interval (CI): 1.0–9.0] to 7.6% (95% CI: 2.5–15) for new hearing loss, and between 4.5% (95% CI: 1.2–15.0) and 14.8% (CI: 6.3–26.1) for new tinnitus following COVID-19. The estimates vary due to use of differing study inclusion-exclusion criteria. More recently, there have been similar reports of audiovestibular symptoms following COVID-19 vaccination (11).

However, caution is required when interpreting these data because the quality of evidence is generally low as most studies have been retrospective surveys, relying on self-report and recall, and lacking a baseline or/and non-COVID control group. As a result, it is difficult to differentiate if symptoms are associated with COVID-19, the vaccine, or issues such as anxiety or reporting bias.

The overall objective of the current study was to make this differentiation by comparing data on auditory and non-auditory symptoms (including a “foil” symptom not established as being associated with COVID-19) reported by individuals prior to, and over the first 18 months of the pandemic (Mar 2020–Sep 2021). This comparison was possible due to the availability of pre-pandemic baseline data on hearing difficulties and tinnitus from a representative cross-section of the UK population collected in March 2019 (12). These same individuals were approached again in September 2021 (during the pandemic) and were asked about the onset and change in six specific symptoms. Three of these symptoms have a known association with COVID-19 (persistent fatigue, loss of smell, problems with memory and concentration) and are referred to below as Type One symptoms. Two of these symptoms (the focus of this paper) have an indeterminate association with COVID-19 (hearing difficulty, tinnitus) and are referred to as Type Two symptoms, and one has no established association with COVID-19 (toothache) and is referred to as a Type Three symptom. The rationale for this was that if hearing difficulty and/or tinnitus are directly associated with COVID-19 illness, the pattern of onset and change would be similar to that seen for the Type One symptoms (persistent fatigue, loss of smell, problems with memory and concentration). Conversely, if hearing difficulty and/or tinnitus are indirectly associated with COVID-19, reflecting psychosocial factors and/or recall bias, then the pattern of onset and change would be more similar to that seen for the Type Three symptom (toothache). The research questions were: (1) Are reports of auditory symptoms associated with COVID-19 status? (2) Does the onset of reported auditory symptoms among individuals with confirmed or probable COVID-19 coincide with their COVID-19 illness? (3) To what extent do individuals attribute auditory and non-auditory symptoms to COVID-19 illness? (4) Is there an interaction between the types of new symptoms reported and (i) COVID-19 status, and (ii) psychosocial challenges encountered during the pandemic, and (5) Are reports of auditory symptoms associated with being vaccinated against COVID-19?

## Materials and Methods

The study was approved by the University of Manchester Research Ethics Committee (Ref: 2020-10483-16733). Informed consent was obtained online as a condition for beginning the survey. The study was preregistered on the Open Science Framework (https://osf.io/35p6t/).

### Participants

Participants were members of the general public aged 18 years or over who lived in the UK and had completed a YouGov survey in March 2019 as part of a larger cross-sectional study—referred to here as Wave One data (12, 13). The sample was representative of the UK population at the time of that survey. YouGov is an internet-based market research company that conducts online surveys using active sampling in which participants invited to complete a survey are selected because they meet specified criteria. Participants who complete a survey are reimbursed in the form of points that accumulate across surveys and can then be exchanged for rewards. Wave Two data collection took place between August 19th and September 13th 2021.

### Survey

A copy of the complete survey can be found in Supplementary Materials File 1. It consisted of four categories of question:

#### COVID-19 Illness and COVID Vaccination

Occurrence and severity of illness, uptake of vaccination.

#### Health and Specific Symptoms

The specific symptoms queried were persistent fatigue, loss of smell, problems with memory and concentration (Type One symptoms), hearing difficulty, tinnitus (Type Two symptoms), and toothache (Type Three symptom). Toothache was chosen as the Type Three symptom following an extensive search of the literature which, at the time the search took place (May 2021), revealed just 2 symptoms that hadn't been reported following COVID-19: toothache and photosensitivity. Toothache was selected because we assumed the general public would be more familiar with the term “toothache” than “photosensitivity.” For each symptom reported, participants were asked about its onset relative to COVID-19/vaccination, changes over time and the extent to which they attributed the symptom to COVID-19 illness.

#### Hearing and Tinnitus

Self-ratings of hearing, tinnitus, help seeking, and use of hearing assistive technology (hearing aids, cochlear implants, etc.).

#### Life Style Questions Focusing on the Prior 12 Months

Life style questions addressed challenges experienced during the pandemic and behavioral approaches taken during the initial lockdown, cigarette smoking, and alcohol consumption. Responses to the latter questions will be addressed in a future publication.

Questionnaire items were presented using branching logic tailored to each participant's individual responses. With the exception of a final question asking whether participants wanted to share additional information, all questions were presented in a multiple choice format.

### Procedure

Emails with an invite to participate were sent during August 2021 by YouGov to all 10,401 participants that had completed Wave One in March 2019. Participants that had not responded within 2 weeks were sent a follow-up message. Fully anonymised data were sent via email as an SPSS data file (SAV) to researchers at the University of Manchester where they were analyzed.

### Analyses

The data were analyzed using IBM SPSS Statistics 25 (IBM Corp, 2017). Descriptive summaries, analyses of variance and chi-square tests were used to analyse responses, with *post-hoc* testing conducted using Bonferroni corrections to adjust for multiple comparisons.

## Results

### COVID-19, Vaccination Status and Demographic Information

Complete surveys were obtained from 66.2% (*n* = 6,881) of the 10,401 individuals who completed Wave One. Six percent of respondents (*n* = 411) reported COVID-19 confirmed by a positive test (COVID+), with a further 11% (*n* = 760) reporting they had probably had COVID-19 (COVID-P). The remaining 83% (*n* = 5,710) reported not having had COVID-19 (COVID-0). It is probable that some COVID-P respondents did not have COVID-19, and that some COVID-0 respondents had asymptomatic COVID-19.

Table 1 shows age at Wave One, gender and ethnicity for all participants combined, and split by COVID status. COVID-0 participants were significantly older than COVID+ and COVID-P participants by around 8 years. This could be due the earlier availability of the vaccine for older individuals in the UK.

**Table 1:** Demographic information for all participants, and split by COVID-19 status.

		**All (*n* = 6,881)**	**^**a**^COVID+ (*n* = 411)**	**^**b**^COVID-P (*n* = 760)**	**^**c**^COVID-0 (*n* = 5,710)**	**Between-group comparison**
Age (yr.)	Mean	52.1	45.4	46.4	53.3	*F* = 102.4
	(SD)	(16.1)	(15.2)	(14.6)	(16.1)	*p* < 0.001
Gender	Female	53.2	51.8	53.9	53.2	χ^2^ = 8.52
	Male	46.6	47.9	45.8	46.6	*p* = 0.202
	Other	0.0	0.2	0.0	0.0	
	^d^NR	0.2	0.0	0.3	0.2	
Ethnicity	White	95.3	94.2	93.4	95.8	χ^2^ = 19.92
	Mixed	1.0	0.7	1.9	0.9	*p* = 0.011
	Asian	2.0	3.2	2.7	1.7	
	Black	0.6	0.2	1.1	0.6	
	Other	0.3	0.7	0.1	0.3	
	^d^NR	0.8	1.0	0.9	0.8	

*Age in years. For other variables value shown is percentage within COVID status*.

a *COVID-19 confirmed by a positive test*;

b *Probable COVID-19 by self-report*;

c *No COVID-19 by self-report*;

d *No response*.

### Hearing Difficulty, Tinnitus and COVID-19

Of the 6,881 Wave Two respondents, 10.2% (*n* = 701) reported both hearing difficulty and tinnitus, 8.8% (*n* = 602) hearing difficulty only, 11.7% (*n* = 802) tinnitus only, 64.5% (*n* = 4,439) neither, and 4.9% (*n* = 337) unsure.

### Is the Reporting of Auditory Symptoms Associated With COVID-19 Status?

In order to assess whether reported auditory symptoms differed by COVID-19 status (COVID+, COVID-P, COVID-0) we first focused on individuals reporting new auditory symptoms in the Wave Two survey, i.e., no reported hearing difficulties and/or tinnitus at Wave One (Table 2). Around 1 in 8 with confirmed or probable COVID-19 reported new hearing difficulty compared to 1 in 15 with no COVID-19. Chi-squared testing showed a significant main effect of COVID status (χ^2^ = 50.6, *p* < 0.001), and paired comparisons with Bonferroni corrections showed significantly less reported hearing difficulty by COVID-0 individuals than for the other two groups (*p* < 0.05). Similarly for tinnitus, about 1 in 6 individuals with confirmed or probable COVID-19 reported new tinnitus compared with 1 in 12 with no COVID-19. Again there was a significant main effect of COVID status (χ^2^ = 50.0, *p* < 0.001), with significantly less tinnitus reported by the COVID-0 group than the COVID+ and COVID-P groups (*p* < 0.05). Reported auditory symptom(s) did not differ between the COVID+ and COVID-P groups (*p* > 0.05).

**Table 2:** Percentage and number (in brackets) of respondents reporting hearing difficulties (*n* = 5,067) and/or tinnitus (*n* = 5,435) in Wave Two who did not report these in Wave One.

	**Have you had COVID?**		
	**^**a**^COVID+**	**^**b**^COVID-P**	**^**c**^COVID-0**
**Have you had hearing difficulty?**			
Yes	12.5% (*n* = 36)	15.1% (*n* = 78)	6.8% (*n* = 277)
No	87.5% (*n* = 252)	84.9% (*n* = 439)	93.2% (*n* = 3,782)
**Have you had tinnitus?**			
Yes	16.3% (*n* = 50)	16.6% (*n* = 90)	8.6% (*n* = 375)
No	83.7% (*n* = 256)	83.4% (*n* = 452)	91.4% (*n* = 3,989)

a *COVID-19 confirmed by a positive test*;

b *probable COVID-19 by self-report*;

c *No COVID-19 by self-report*.

Change in auditory symptoms by COVID-19 status was also examined. Individuals who at Wave Two reported that their hearing difficulties (*n* = 1,111) and/or tinnitus (*n* = 1,274) had begun prior to the pandemic were asked whether their symptom(s) had changed since the pandemic had begun. Fourteen percent said their hearing had become worse, and 3.6% said their hearing had improved. The equivalent numbers for changes in tinnitus symptoms were 11.5, and 4.6%, respectively.

Table 3 shows the associations between changes in auditory symptoms and COVID status. Although the majority of participants reported no changes in their auditory symptom(s), significantly more COVID+ and COVID-P individuals reported a deterioration than COVID-0 (*p* < 0.05). A minority of individuals reported improvements in their auditory symptoms however, these findings should be interpreted with caution as the numbers of individuals in each group are small.

**Table 3:** Percentage and number (in brackets) of reported changes in hearing since the start of the pandemic for people reporting in the Wave Two survey that their hearing difficulties (*n* = 1,111) and/or tinnitus (*n* = 1,274) began prior to the pandemic.

	**Have you had COVID?**			**Between-group comparison**
	**^**a**^COVID+**	**^**b**^COVID-P**	**^**c**^COVID-0**	
**Have there been any changes in your hearing since the pandemic began?**				
It has got worse	30.8% (*n* = 12)	27.3% (*n* = 27)	11.9% (*n* = 116)	χ^2^ = 57.1, *p* < 0.001
No change	64.1% (*n* = 25)	58.6% (*n* = 58)	84.7% (*n* = 824)	
It has improved	5.1% (*n* = 2)	12.1% (*n* = 12)	2.7% (*n* = 26)	
Don't know/can't recall	0.0% (*n* = 0)	2.0% (*n* = 2)	0.7% (*n* = 7)	
**Have there been any changes in your tinnitus since the pandemic began?**				
It has got worse	23.1% (*n* = 12)	22.8% (*n* = 29)	9.7% (*n* = 106)	χ^2^ = 94.8, *p* < 0.001
No change	73.1% (*n* = 38)	57.5% (*n* = 73)	86.6% (*n* = 948)	
It has improved	1.9% (*n* = 1)	18.1% (*n* = 23)	3.1% (*n* = 34)	
Don't know/can't recall	1.9% (*n* = 1)	1.6% (*n* = 2)	0.6% (*n* = 7)	

a *COVID-19 confirmed by a positive test*;

b *probable COVID-19 by self-report*;

c *No COVID-19 by self-report*.

To summarize, while these data show more reports of new auditory symptoms and more reports of worsened auditory symptoms among the COVID-+ and COVID-P respondents than COVID-0 respondents, it is important to consider the findings below which indicate that additional factors influence symptom reporting.

### Does the Onset of Auditory Symptoms Among Individuals With Confirmed or Probable COVID-19 Coincide With Their COVID-19 Illness?

Reported onset of new auditory symptoms since Wave One is shown in Table 4. Around 1 in 3 respondents in the COVID+ and COVID–P groups said their auditory symptoms began when they got ill with, or within a few months of, COVID-19. Most of the other individuals either could not recall when their symptoms had begun or said the symptoms had begun before the pandemic—which is surprising because they had not reported these in Wave One.

**Table 4:** Percentage and number (in brackets) of respondents reporting onset of hearing difficulties (*n* = 594) and tinnitus (*n* = 738) by COVID-19 status for respondents not reporting hearing difficulties in the Wave One survey.

	**Have you had COVID?**			
	**^**a**^COVID+**	**^**b**^COVID-P**	**^**c**^COVID-0**	**Total**
**When did your hearing difficulty begin?**				
Before March 2020	17.5% (*n* = 11)	29.0% (*n* = 36)	50.9% (*n* = 207)	42.8% (*n* = 254)
When I got ill with COVID/within a few months of having COVID	34.9% (*n* = 22)	30.6% (*n* = 38)	NA	10.1% (*n* = 60)
During the pandemic but not because of COVID	4.8% (*n* = 3)	3.2% (*n* = 4)	17.2% (*n* = 70)	13.0% (*n* = 77)
Don't know/can't recall	42.9% (*n* = 27)	37.1% (*n* = 46)	31.9% (*n* = 130)	34.2% (*n* = 203)
**When did your tinnitus begin?**				
Before March 2020	25.0% (*n* = 18)	28.9% (*n* = 43)	53.6% (*n* = 277)	45.8% (*n* = 338)
When I got ill with COVID/within a few months of having COVID	38.9% (*n* = 28)	26.8% (*n* = 40)	NA	9.2% (*n* = 68)
During the pandemic but not because of COVID	5.6% (*n* = 4)	4.7% (*n* = 7)	19.0% (*n* = 98)	14.8% (*n* = 109)
Don't know/can't recall	30.6% (*n* = 22)	39.6% (*n* = 59)	27.5% (*n* = 142)	30.2% (*n* = 223)

a *COVID-19 confirmed by a positive test*;

b *probable COVID-19 by self-report*;

c *No COVID-19 by self-report*.

In terms of onset, then, auditory symptoms coincided with COVID-19 illness in about 1/3 of participants with confirmed or probable COVID-19.

### To What Extent Do Individuals Attribute Auditory and Non-auditory Symptoms to COVID-19 Illness?

Participants were asked to rate the extent to which they think catching COVID-19 had affected each symptom that was reported to have begun when they got ill with COVID/within a few months of having COVID. Table 5 shows these results. Unsurprisingly, over 80% of respondents said their Type One symptoms were affected “somewhat” or “very much” by COVID-19. Over 65% said their hearing difficulties and/or tinnitus were “somewhat” or “very much” affected by COVID-19 and unexpectedly, almost as many said their toothache was “somewhat” or “very much” affected by COVID-19. Further, 57% of the individuals who reported both hearing difficulty and toothache (*n* = 37) said both were “somewhat” or “very much” affected by COVID-19, as did 73% of the individuals who reported both tinnitus and toothache (*n* = 37).

**Table 5:** Effects of catching COVID-19 on each symptom reported to have begun when the individual got ill with COVID/within a few months of having COVID.

	**Effects of catching COVID-19 on each symptom report**				
**Symptom**	**Not at all**	**A little**	**Somewhat**	**Very much**	**Don't know**
Persistent fatigue (*n* = 514)	1.4	9.7	17.1	68.9	2.9
Loss of smell (*n* = 369)	3.3	8.7	12.7	73.2	2.2
Problems with memory/ concentration (*n* = 302)	1.3	12.9	28.1	54.0	3.6
Hearing difficulty (*n* = 101)	5.9	20.8	25.7	40.6	6.9
Tinnitus (*n* = 104)	6.7	10.6	28.8	42.3	11.5
Toothache (*n* = 76)	5.3	21.1	30.3	30.3	13.2

*Value shown is percentage for each symptom*.

In other words, both auditory and non-auditory symptoms were attributed to COVID-19, even in the absence of established evidence linking them.

### Is There an Interaction Between the Types of New Symptoms Reported and COVID-19 Status?

It is important to consider the reporting and onset of the non-auditory symptoms to determine if there is an interaction between COVID status and reporting by symptom type. To this end, we examined the proportion of all Wave Two participants reporting one or more of each symptom type (new or pre-existing) relative to COVID-19 status (Table 6).

**Table 6:** Percentage of participants reporting one or more of each type of symptom, in Wave Two, relative to COVID-19 status.

		**Have you had COVID-19?**			**Between-group comparison**
**Reports of one or more:**	**Response**	**^**a**^COVID+ (*n* = 411)**	**^**b**^COVID-P (*n* = 760)**	**^**c**^COVID-0 (*n* = 5,710)**	
^d^Type One symptoms	Yes	84.7	76.8	33.9	χ^2^ = 872.4
	No	13.9	20.0	64.4	*p* < 0.001
	Unsure	1.5	3.2	1.7	
^e^Type Two symptoms	Yes	18.7	23.3	19.1	χ^2^ = 44.1
	No	78.3	74.3	80.2	*p* < 0.001
	Unsure	2.9	2.4	0.7	
^f^Type Three symptom	Yes	12.4	17.1	12.1	χ^2^ = 78.4
	No	82.0	74.5	84.9	*p* < 0.001
	Unsure	5.6	8.4	3.0	

a *COVID-19 confirmed by a positive test*;

b *probable COVID-19 by self-report*;

c *No COVID-19 by self-report*;

d *Persistent fatigue, Loss of smell, Problems with memory or concentration*;

e *Hearing difficulty, Tinnitus*;

f *Toothache*.

Reporting of Type One symptoms followed the expected pattern such that these were reported most by the COVID+ group, followed by the COVID-P and COVID-0 groups. All three groups differed significantly from one another (*p* < 0.05).

Type Two and Type Three symptoms, however, followed a different pattern, such that the COVID-P group reported these symptoms the most (*p* < 0.05 for COVID-P vs. COVID-0 for Type Two and Type Three symptoms). Equally interestingly, no differences in rates of reporting were found between the COVID+ and COVID-0 groups. This differs from the data in Table 2 because here we include all participants (not just those with new auditory symptoms) and because the data in Table 2 are separated by type of auditory symptom while here they are combined.

To summarize, (i) the COVID+ group reported the most Type One symptoms, (ii) COVID+ and COVID-0 groups did not differ in their reporting of Type Two and Type Three symptoms, and (iii) COVID-P individuals reported more Type Two and Type Three symptoms than COVID+ and COVID-0 individuals.

### Is There an Interaction Between the Types of New Symptoms Reported and Psychosocial Challenges Encountered During the Pandemic?

About 1 in 3 participants reported mental health challenges during the pandemic (e.g., anxiety, loneliness, feeling down), physical health challenges (lack of exercise, unusual aches and pains), and challenges adjusting to a changed daily routine. Around 1 in 10 stated care commitment challenges (childcare, looking after elderly relatives), environmental challenges (lack of space at home, no access to outside space), financial challenges, and employment challenges. One in 25 reported other types of challenges. About 1 in 3 noted no such challenges during the pandemic.

Table 7 shows the association between symptom type and the number of challenges encountered (none, 1 or 2, >2). It can be seen that people who encountered more psychosocial challenges also reported more symptoms. This was the case for all symptom types.

**Table 7:** Percentage of participants reporting one or more of each type of symptom relative to number of challenges experienced during the pandemic.

		**Challenges encountered**			**Between-group comparison**
**Reports of one or more:**	**Response**	**0 (*n* = 2359)**	**1–2 (*n* = 2928)**	**3+ (*n* = 1594)**	
^a^Type One symptoms	Yes	26.2	42.2	63.6	χ^2^ = 569.3
	No	70.4	56.2	36.2	*p* < 0.001
	Unsure	3.4	1.5	0.3	
^b^Type Two symptoms	Yes	16.4	19.7	23.9	χ^2^ = 52.2
	No	82.9	79.5	74.2	*p* < 0.001
	Unsure	0.7	0.8	1.9	
^c^Type Three symptom	Yes	8.2	13.4	17.9	χ^2^ = 91.5
	No	87.0	83.4	78.8	*p* < 0.001
	Unsure	4.8	3.2	3.3	

a *Persistent fatigue, Loss of smell, Problems with memory or concentration*;

b *Hearing difficulty, Tinnitus*;

c *Toothache*.

To summarize, more symptoms, regardless of type, were reported by individuals encountering more psychosocial challenges.

### Are Reports of Auditory Symptoms Associated With Being Vaccinated Against COVID-19?

Over 93% (*n* = 6,429) of participants had received at least one dose of COVID-19 vaccination (90.4% two doses and 3.0% one dose). The proportions having Oxford/AstraZeneca, Pfizer/BionTech vaccine and Moderna were 57.8, 38.8, and 2.1%, respectively, with 1.4% having a different vaccine or not knowing which vaccine they had received.

Of the 4,687 vaccinated respondents who did not report hearing difficulty at Wave One, 1.2% (*n* = 57) reported hearing difficulty at some point following vaccination. Of these 23 said the hearing difficulties began within a week of the vaccination, and 34 said the hearing difficulties began more than a week after the vaccination. For tinnitus, of the 5,068 vaccinated respondents who did not report tinnitus at Wave One, 1.5% (*n* = 78) reported tinnitus at some point following vaccination, with 31 reporting tinnitus onset within a week of vaccination and 47 reporting tinnitus onset more than a week after vaccination.

Regarding vaccine type, hearing difficulties following vaccination were reported by 1.82, 0.97, and 1.49% of recipients of the Moderna, Oxford/AstraZeneca, and Pfizer/BionTech vaccines, respectively (χ^2^ = 26.3, *p* < 0.002). Equivalent data for reports of tinnitus are 1.88, 1.78, and 1.26% (χ^2^ = 10.1, *p* = 0.341). Caution is needed when interpreting these findings as there are few reports of auditory symptoms following vaccination.

When the effect of the vaccination on auditory symptoms is examined in terms of change relative to Wave One reports, about 90% of respondents reported no change in their hearing and/or tinnitus, about 2% said their hearing and/or tinnitus had improved, 6.7% said their hearing had become worse, and 4.7% said their tinnitus had become worse.

There were significant interactions between auditory symptoms reported and number of vaccination doses received, such that more individuals who had one dose rather than two doses reported new hearing difficulty (5.4 vs. 1.1%, *p* < 0.001) and new tinnitus (3.7 vs. 1.5%, *p* < 0.041) as a result of the vaccination.

These low rates of reported auditory symptoms following COVID-19 vaccination suggests that COVID-19 vaccination has little or no impact on the auditory system.

## Discussion

This study aimed to disentangle the direct associations between COVID-19 and auditory symptoms (hearing difficulty and tinnitus) from the indirect associations due to inconsistent reporting, recall bias, and psychosocial factors. We achieved this by comparing auditory symptoms reported in a 2019 survey (pre-pandemic) with auditory and other symptoms reported in the 2021 survey (during the pandemic). In the latter, we examined the onset of three types of symptoms as a function of COVID-19 status—known association with COVID-19 (Type One), indeterminate association with COVID-19 (the auditory symptoms, Type Two) and one with no established association with COVID-19 (Type Three).

Our first research question asked whether reports of auditory symptoms were associated with COVID-19 status. Indeed, we found a 2-fold increase in reported auditory symptoms among people with confirmed or probable COVID-19, relative to those not reporting COVID-19. The difference between the COVID-0 and COVID + groups was 4.7% for new hearing difficulties and 7.7% for new tinnitus—values which fall within the rates revealed by meta-analyses (6, 9, 10).

When examined in terms of change in auditory symptoms during the pandemic, we learned that a deterioration in hearing was reported significantly more often by people with confirmed or probable COVID-19 than by those without COVID-19—again consistent with an association between COVID-19 and auditory symptoms.

A minority of individuals reported improvements in their auditory symptoms during the pandemic. This might be explained by life style changes leading to quieter surroundings and a decreased need to communicate in difficult (noisy) listening environments (14–16); transient hearing difficulties associated with cerumen blockage, otitis media, and middle ear fluid from colds or allergies, etc.; and/or more time to engage in activities such as relaxation, mindfulness and exercise that are known to help with tinnitus management (17).

While the above data generally indicate increased reporting of auditory symptoms among people with confirmed or probable COVID-19, other findings suggest the situation is more nuanced.

First, new auditory symptoms coincided with COVID-19 illness among just one third of COVID+ and COVID-P individuals, with another third not being able to recall when their symptoms began, and a third saying their symptoms started before the pandemic—despite reporting no such symptoms at Wave One. Regarding this final point, it is highly improbable that the symptoms of so many individuals could have started in the 12-month period between completion of the Wave One survey and the start of the pandemic because it is known that the annual incidence of tinnitus is 1–2% (18, 19). Inconsistent recall or reporting bias is more likely. One explanation is that self-report is simply unreliable, another is that participants interpreted the two questions asking about hearing difficulty and tinnitus differently from survey to survey because of the context in which they were asked. Specifically, in Wave One the focus was on perception of personal risk of key health conditions, which could have led to underreporting of symptoms. In Wave Two, the focus was on symptoms experienced during the pandemic, which could have led to increased reporting of symptoms. Indeed, the influence of the context in which a question is asked has been shown to influence symptom reporting (20–22).

Second, over 60% of individuals with confirmed or probable COVID-19 said their toothache had been affected by their being ill with COVID-19, despite a lack of established evidence that toothache is associated with COVID-19. Moreover, there was considerable overlap between the individuals who attributed their hearing difficulty (*n* = 37) and/or tinnitus (*n* = 37) to COVID-19 and who attributed their toothache to COVID-19 (57% and 73%, respectively). We cannot determine whether this is because these symptoms are indeed associated with COVID-19 or whether they reflect a nocebo effect, i.e., new or worsening symptoms that develop in response to negative health-related information, beliefs, and/or experiences (23). Such effects have been shown in response to medications (24, 25), vaccinations (26), and, as most relevant here, public health scares (27, 28).

Third, we found that COVID status interacted with the types of symptoms reported. As expected, the COVID + group reported the most Type One symptoms (i.e., symptoms with a known association with COVID-19). Fewer of these symptoms were reported by the COVID-P group, presumably because some individuals in this group did not in fact have COVID-19. The fewest Type One symptoms were reported by the COVID-0 group, although still at a relatively high rate (34%), which again, might be the context in which the question was asked.

However, it is important to recall that, for Type 2 and 3 symptoms, reports of auditory symptoms are similar in the COVID+ (confirmed cases) and COVID-0 groups. This is in agreement with a recent study (29) that reported similar proportions of auditory symptoms in COVID cases and non-COVID controls. Comparator groups are a fundamental design characteristic of high quality clinical trials which are lacking in the majority of studies in the systematic reviews summarized in the introduction. As illustrated by our findings, the results of studies that lack a comparator groups should be interpreted with caution. On the other hand, the COVID-P group reported the highest levels of Type Two and Type Three symptoms which raises a question as to why this group of individuals, who have unconfirmed COVID-19, also report more symptoms with unestablished links with COVID-19 than either of the other COVID groups. It might be that some individuals in this group are highly sensitive to somatic sensations and health anxiety leading to heightened symptom perception and reporting (30).

The complexity of symptom perception and reporting is further illustrated in our data showing a positive association between reporting of symptoms and the number of reported psychosocial challenges encountered during the pandemic. There are at least three explanations for this finding. First, it might be that the stress of psychosocial challenges resulted in illness or raised awareness of symptoms and thus increased symptom reporting. Second, it might be that being ill led to psychosocial challenges, or third, that some other factor, such as personality, heightened the awareness of both psychosocial challenges and symptoms—hence the positive association. There has long been awareness that such relationships are complex and that there is considerable individual variability in how they manifest (31–33).

Finally, we examined whether our data provided evidence that the COVID-19 vaccination impacts the auditory system. There were few reports of new and/or worsened auditory symptoms following vaccination, at rates were lower than the reports of auditory symptoms among the COVID-0 group and in similar proportions across vaccine type. This suggests little or no association between auditory symptoms and COVID-19. Interestingly, however, more auditory symptoms were reported by individuals who had one, rather than two, doses of vaccine. We interpret this to indicate that individuals who perceived symptoms after one dose decided not to pursue a second dose.

## Conclusions

We conclude that despite there being more reports of auditory symptoms from individuals with confirmed or probable COVID-19, there is evidence of inconsistent reporting and/or recall bias, as well as possible nocebo effects. Studies that include appropriate control groups and use audiometric measures in addition to self-report to investigate change in auditory symptoms relative to pre-COVID-19 are urgently needed.

## Data Availability Statement

The datasets presented in this article are not readily available because Ethics approval was not requested or granted. Requests to access the dataset should be directed to Gabrielle H. Saunders, gabrielle.saunders@manchester.ac.uk.

## Ethics Statement

The studies involving human participants were reviewed and approved by University of Manchester Research Ethics Committee Ref: 2020-10483-16733. Written informed consent for participation was not required for this study in accordance with the national legislation and the institutional requirements.

## Author Contributions

GS, EB, KU, CA, and KM contributed to conception and design of the study. GS and JK performed the statistical analysis. GS wrote the first draft of the manuscript. KM wrote sections of the manuscript. All authors contributed to the article and approved the submitted version.

## Funding

This research was funded by the NIHR Manchester Biomedical Research Centre, British Tinnitus Association, NeuroMod Devices Ltd., and the Royal National Institute for Deaf People (RNID).

## Conflict of Interest

The authors declare that the research was conducted in the absence of any commercial or financial relationships that could be construed as a potential conflict of interest.

## Publisher's Note

All claims expressed in this article are solely those of the authors and do not necessarily represent those of their affiliated organizations, or those of the publisher, the editors and the reviewers. Any product that may be evaluated in this article, or claim that may be made by its manufacturer, is not guaranteed or endorsed by the publisher.
