# Supplementary material for: Shedding Light on SARS-CoV-2, COVID-19, COVID-19 Vaccination, and Auditory Symptoms: Causality or Spurious Conjunction?
Source: Front Public Health. 2022 Feb 22;10:837513. doi: 10.3389/fpubh.2022.837513 (PMC8919951; doi:10.3389/fpubh.2022.837513)
Supplement: Supplementary file 1 [file Data_Sheet_1.docx]

**SOME QUESTIONS ABOUT YOU**

**1. Gender**

[Gender_2] Which of the following describes how you think of yourself?

<1>Male

<2>Female

<3>In another way

<97> Prefer not to say

**2. Age (in years)** (Drop down menu in individual age)

**3. What ethnic group best describes you? Please select one option only.**

<1> English / Welsh / Scottish / Northern Irish / British

<2> Irish

<3> Gypsy or Irish Traveller

<4> Any other White background

<5> White and Black Caribbean

<6> White and Black African

<7> White and Asian

<8> Any other Mixed / Multiple ethnic background

<9> Indian

<10> Pakistani

<11> Bangladeshi

<12> Chinese

<13> Any other Asian background

<14> African

<15> Caribbean

<16> Any other Black / African / Caribbean background

<17> Arab

<18 fixed> Any other ethnic group

<19 fixed> Prefer not to say

*Contingencies and contingent items in italics and green highlights*

**SOME QUESTIONS ABOUT COVID-19**

**4. Have you had COVID-19?**

- Yes – confirmed by a positive test
- Probably – but not confirmed by a test
- Don’t know
- No

*For participants who said ‘yes’ or ‘probably’ to Q4 (reported having had COVID):*

How was your COVID or suspected COVID primarily managed?

At home

In hospital not ICU

In ICU

*Questions for all*

**5. We want to know a bit about your health and some symptoms you may have had.** Put a tick in the box that describes whether you have each symptom and approximately when each began.

*Note: Include columns 4, 5 and 6 only if respondent said ‘yes’ or ‘probably’ to Q4 (reported having had COVID).*

| Symptom | Not had this symptom | **Began** before March 2020 (i.e. before the pandemic) | **Began** when I got ill with COVID | **Began** a few weeks after I had COVID | **Began** a few months after I had COVID | **Began** during the pandemic but not because I had COVID | Don’t know / can’t recall |
| --- | --- | --- | --- | --- | --- | --- | --- |
| Persistent fatigue (constantly feeling tired and lacking energy even after rest) |  |  |  |  |  |  |  |
| Loss of smell |  |  |  |  |  |  |  |
| Tinnitus (noises in the head or ears lasting more than 5 min) |  |  |  |  |  |  |  |
| Toothache |  |  |  |  |  |  |  |
| Problems with memory and concentration (‘Brain fog’) |  |  |  |  |  |  |  |
| Difficulty hearing |  |  |  |  |  |  |  |

*For conditions reported to have existed before the pandemic: (symptoms checked in Q5 column 3)*

**6. Have there been any changes in these symptoms since the pandemic began?**

| Symptom | It has got worse | No change | It has improved | Don’t know / can’t recall |
| --- | --- | --- | --- | --- |
| *Include symptoms checked in Q6 column 3* |  |  |  |  |
| *i.e. present before the pandemic* |  |  |  |  |
|  |  |  |  |  |

*For people reporting ‘yes’ or ‘probably’ to Q4 (had COVID), ask about symptoms that began as a result of having COVID (checked in Q5 columns 4, 5 and/or 6):*

**7. How long did your COVID symptoms last?**

| Symptom | Lasted only while I was ill with COVID | Lasted for about a month | Lasted for more than a month but less than 3 months | Lasted for longer than 3 months | The symptom started so recently that I don’t know |
| --- | --- | --- | --- | --- | --- |
| *Include symptoms checked in Q5 columns 4, 5, and/or 6* |  |  |  |  |  |

*For people reporting ‘yes’ or ‘probably’ to Q4 (had COVID), ask about symptoms that began as a result of having COVID (checked in Q5 columns 4, 5 and/or 6):*

**8. How much do you think catching COVID-19 affected the following symptoms?**

| Symptom | Not at all | A little | Somewhat | Very much | Don’t know |
| --- | --- | --- | --- | --- | --- |
| *Include symptoms checked in Q5 columns 4, 5, and/or 6* |  |  |  |  |  |

*Questions for all*

**QUESTIONS ABOUT COVID-19 VACCINATIONS**

**9. Have you had a COVID vaccination?**

☐ Yes one dose

☐ Yes two doses

☐ No

*If yes to q9 (had a vaccination):*

**9a. Which vaccination did you have? (pull down list, include option for ‘unsure’)**

Moderna vaccine

Oxford / AstraZeneca vaccine

Pfizer / BioNTech vaccine

Don’t know / other

**9b. Did you have any of the following symptoms after the vaccination?**

|  | Not had this following my vaccination | Yes, it began within a week of my vaccination | Yes, it began more than a week after I had the vaccination | Don’t know / can’t recall |
| --- | --- | --- | --- | --- |
| Fatigue |  |  |  |  |
| Loss of smell |  |  |  |  |
| Tinnitus (noises in the head or ears) |  |  |  |  |
| Toothache |  |  |  |  |
| Problems with memory and concentration (‘Brain fog’) |  |  |  |  |
| Difficulty hearing |  |  |  |  |

*For symptoms reported to have begun as a result of the vaccination (Q9b columns 3 & 4):*

**9c. How long did these symptoms last?**

| Symptom | Only lasted for a few hours after the COVID-19 vaccination | Lasted for a few days after the COVID-19 vaccination | Lasted for a couple of weeks after the COVID-19 vaccination | | Lasted longer than 4 weeks after the COVID-19 vaccination | I was vaccinated so recently that I don’t yet know | Don’t know / can’t recall |
| --- | --- | --- | --- | --- | --- | --- | --- |
| *Include symptoms checked in Q9b columns 3 and 4* |  |  | |  |  |  |  |

*For people who have had a vaccination (yes to Q9) and for symptoms reported to have existed prior to the pandemic (checked in Q5 column 3)*

**9d. Have any of these symptoms changed since you had your COVID-19 vaccination?**

|  | It has got worse | No change | It has improved | Don’t know / can’t recall |
| --- | --- | --- | --- | --- |
| *Include symptoms checked in Q5 columns 3* |  |  |  |  |

*For participants who have had a COVID-19 vaccination (yes to Q9) and who reported symptoms that started after their vaccination (checked in Q9b, columns 3 and 4).*

**9e. How much do you think the COVID-19 vaccine affected the following symptoms?**

| Symptom | Not at all | A little | Somewhat | Very much | Don’t know |
| --- | --- | --- | --- | --- | --- |
| *Include symptoms checked in Q8b columns 3 and 4* |  |  |  |  |  |

**QUESTIONS ABOUT YOUR TINNITUS AND HEARING**

*For people answering they have tinnitus (checked in Q5 line 4, columns, 3, 4, 5, 6 or 7 or Q9b line 4 columns 3 or 4)*

**10a. Where do you notice your tinnitus?**

- Right ear only
- Left ear only
- Both ears
- In my head
- Don’t know
- Other please specify

**10b. Which best describes your tinnitus**

- It is there most of the time
- It is there some of the time
- I don’t notice it very often

**10c. How bothersome, if at all, is your tinnitus?**

- Not at all
- Mildly bothersome
- Moderately bothersome
- Very bothersome
- Extremely bothersome

**10d. Have you ever told a doctor or nurse about your tinnitus?**

- Yes
- No

*Questions for all*

**11. Do you have any difficulty with your hearing?**

- Yes
- No

*Hearing rating Qs from ELSA*

**12. My hearing is:**

- Excellent
- Very good
- Good
- Fair
- Poor

**13. How much difficulty do you have following a conversation if there is background noise?**

- Slight difficulty
- Moderate difficulty
- Great difficulty

**14. Have you ever told a doctor or nurse about your hearing problems?**

- Yes
- No

**15. Do you have and devices that help you hear?** (tick all that apply)

- No
- Hearing aid(s)
- Cochlear implant(s)
- Bone anchored hearing aid(s)
- Other
  - What ______________

*If has a hearing device (yes to Q15)*

**15a. How often do you use your hearing device(s)?**

- Most of the time
- Some of the time
- Not at all

**SOME QUESTIONS ABOUT YOUR LIFESTYLE**

**16. Which of the following best describes you?**

- I have never smoked a cigarette, not even a puff
- I have never really smoked but I have tried a few puffs
- I used to smoke but now I have stopped
- I don’t smoke every day but every week (or from time to time)
- I smoke every day

**17. How often have you had an alcoholic drink during the last 12 months?**

- Almost every day
- 5 or 6 times a week
- 3 or 4 times a week
- Once or twice a week
- Once or twice a month
- Once every couple of months
- Once or twice over the year
- Not at all in last 12 months

**18. Which, if any, of the following challenges have you faced since the start of the COVID-19 pandemic (e.g. March 2020)? Please select all that apply.’**

- Mental health challenges (e.g. anxiety, feeling down, loneliness etc.)
- Physical health challenges (e.g. lack of exercise, unusual aches and pains etc.)
- Adjusting to changes to your usual daily routine
- Care commitment challenges (e.g. childcare or looking after elderly relatives)
- Environmental challenges (e.g. lack of space at home, no access to outside space etc.)
- Financial challenges (e.g. paying bills, rent, accessing the benefit system etc.
- Employment challenges (e.g. being unable to work, being made redundant, furlough etc.)
- Other [open] (please specify)
- N/A – I haven’t faced any challenges since the start of the COVID-19 pandemic
- Prefer not to say

**During the COVID pandemic, some people have been identified as**[**clinically extremely vulnerable**](https://gbr01.safelinks.protection.outlook.com/?url=https%3A%2F%2Fwww.gov.uk%2Fgovernment%2Fpublications%2Fguidance-on-shielding-and-protecting-extremely-vulnerable-persons-from-covid-19%2Fguidance-on-shielding-and-protecting-extremely-vulnerable-persons-from-covid-19%23cev&data=04%7C01%7Ckate.gosschalk%40yougov.com%7C412f531b445e48379dda08d946b04e7c%7C8b848903971d4adcb4764d390db6565b%7C1%7C0%7C637618545447745867%7CUnknown%7CTWFpbGZsb3d8eyJWIjoiMC4wLjAwMDAiLCJQIjoiV2luMzIiLCJBTiI6Ik1haWwiLCJXVCI6Mn0%3D%7C1000&sdata=1kwCbAlu8gymwLENbHwSBHRJGT%2FRWCICt5Wt56QRkOU%3D&reserved=0)**. If you are in this group, you will previously have received a letter from the NHS or from your GP telling you this. You may have been advised to shield in the past. Have you been told to shield at any point since March 2020**?      Yes/No

**19. Please select the main approach you took during the initial COVID-19 lockdown in 2020.**

- Self-isolated due to being at higher risk of contracting the virus (due to other illnesses or older age)
- Self-isolated due to fear of contracting the virus (no social interactions outside your household)
- Rigorously followed government advice about physical distancing, maintaining hand hygiene and wearing face coverings when asked
- Generally followed government advice about physical distancing, maintaining hand hygiene and wearing face coverings when aske
- Made almost no changes to my life during lockdown

**20. If there anything else you would like to tell us about your health during the pandemic or the COVID-19 vaccination please type it in the box below.**
